# Modulation of Immune Components on Stem Cell and Dormancy in Cancer

**DOI:** 10.3390/cells10112826

**Published:** 2021-10-21

**Authors:** Xiaofan Jiang, Lu Liang, Guanglei Chen, Caigang Liu

**Affiliations:** Department of Oncology, Shengjing Hospital, China Medical University, Shenyang 110004, China; 2020122169@cmu.edu.cn (X.J.); luliang4399@163.com (L.L.); glchen@cmu.edu.cn (G.C.)

**Keywords:** cancer stem cell, dormancy, immune cell, intercellular communication, CSC niche

## Abstract

Cancer stem cells (CSCs) refer to a certain subpopulation within the tumor entity that is characterized by restricted cellular proliferation and multipotent differentiation potency. The existence of CSCs has been proven to contribute to the heterogeneity of malignancies, accounting for intensified tumorigenesis, treatment resistance, and metastatic spread. Dormancy was proposed as a reversible state of cancer cells that are temporarily arrested in the cell cycle, possessing several hallmarks that facilitate their survival within a devastating niche. This transient period is evoked to enter an actively proliferating state by multiple regulatory alterations, and one of the most significant and complex factors comes from local and systemic inflammatory reactions and immune components. Although CSCs and dormant cancer cells share several similarities, the clear relationship between these two concepts remains unclear. Thus, the detailed mechanism of immune cells interacting with CSCs and dormant cancer cells also warrants elucidation for prevention of cancer relapse and metastasis. In this review, we summarize recent findings and prospective studies on CSCs and cancer dormancy to conclude the relationship between these two concepts. Furthermore, we aim to outline the mechanism of immune components in interfering with CSCs and dormant cancer cells to provide a theoretical basis for the prevention of relapse and metastasis.

## 1. Introduction

Complete management of cancers remains refractory, due to their several intractable properties, among which the most prominent are intertumoral and intratumoral heterogeneity, which contribute to the complex peculiarity of cancers. The formal approach is now under intense investigation through a more accruable molecular subtyping within each type of malignancy to develop precise treatment regimens, while strategies to manage the latter one seem to be more complicated. For a long time, we have accepted the observation that not all cancer cells are identical, and, in some cancer types, certain cell subpopulations possess a distinguished differentiation degree, and this observation firmly supports the notion that undifferentiated cell proportions are referred to as cancer stem cells (CSCs) [1]. Similar to tissue-specific stem cells, which are indispensable for tissue homeostasis and repair, CSCs play a prominent role in maintaining self-renewal and multipotent differentiation capacity and giving rise to more differentiated cell lineages in cancers [2]. Sufficient evidence has revealed that the presence of CSCs contributes mainly to the heterogeneity of cancers and accounts for several properties that facilitate their survival and aggression, including genotoxic agent resistance [3], radio-resistance [4], oncogenesis [5], metastasis [6], tumor immune-microenvironment remodeling [7], and metabolic reprogramming [8]. Thus, cancer stemness might be the most significant factor for drug resistance, cancer recurrence, and metastasis. Multiple strategies have been proposed to tackle CSCs, including targeting tumor microenvironment, specific cell surface markers, and intrinsic signaling pathways. Many stem-cell surface markers have been well-established, and their expression has also been implicated in the promotion of treatment resistance and cancer progression. For example, the combination of CD44+/CD24− is now commonly employed to define breast-cancer stem cells (BCSC) [9], and most of them are located at the invasive edge, indicating that their presence is associated with enhanced aggression [10]. In addition, expression of CD44 in hepatocellular carcinoma (HCC) promotes sorafenib resistance and tumorigenicity [11]. Aldehyde dehydrogenase (ALDH) is another well-accepted cell-surface marker for BCSCs [12], and studies have reported that ALDH+ BCSCs manifest intensified colony formation, tumor initiation, and chemo-resistance than CD44+/CD24− BCSCs [13]. Other core components of stemness regulatory transcription factors, including Oct4, SOX2, and Nanog, participate in self-renewal and differentiation regulation [14]. These markers involved in cancer stemness may serve as potential cell-intrinsic treatment targets in multiple cancers. However, how cell-extrinsic mutual interaction between tumor immune-microenvironment (TIME) and CSC eradicates cancer cells under immunosurveillance or promotes cancer progression is not yet clear, and elucidating the precise mechanism would assist in harnessing the immune system to effectively recognize and eliminate the possibility of cancer re-emergence or reverse the immunosuppressive niche into a cancer-detrimental one.

Sharing several identical properties with CSC, tumor dormancy has also been proposed as an important contributor to long-term cancer relapse and metastasis, thus accounting for the majority of cancer-related deaths. In many circumstances, patients present with metastases long after comprehensive local and systemic treatment; for instance, the probability of tumor relapse for hormone receptor-positive breast cancer increases steadily for 15 years after completion of 5-year endocrine therapy. This phenomenon was also observed in multiple solid cancers, including lung, colon, and prostate; and hematological malignancies, such as leukemia and multiple myeloma, strongly indicating the persistence of residual cancer cells or minimal residual disease situated in the dormancy state even after effective treatment for cancer. The concept of tumor dormancy is now classified into “population dormancy” and “cellular dormancy”. “Population dormancy” could be further divided into “angiogenic dormancy” and “immune dormancy”, implicating that deficiency in nutrient supplementation and pressure from immunosurveillance induce the proliferation and apoptosis within the residual tumor mass to reach equilibrium, thus the tumor mass maintains to be stable in size and stops progressing. However, in this review, we emphasize the concept of cancer-cell dormancy, in which cells are temporarily and reversibly arrested at the G0/G1 cell-cycle phase. Mounting evidence has revealed that early in the process of tumor initiation, single cancer cells or cell clusters could detach from the primary foci and enter the peripheral circulation in the form of circulating tumor cells (CTCs). Early in this phase, CTCs could adopt phenotypic, genetic, and functional mutations to enter into the dormant state and survive the attack from physical shear force and immunosurveillance [15]. Once they arrive and reside at specific sites of the target organ, such as the endosteal surface of the bone [16,17], CTCs become disseminated tumor cells (DTC) after a series of continuous adaptive regulatory processes, including occupancy of the metastatic niche, interaction and engagement with niche, adaptation to niche through reprogramming, and establishment of long-term dormancy [18]. Cancer cells in dormant state are a “double-edge sword”, because their presence exposes the limited threat instantly, while forced stimulation intrinsically or from niche alteration could result in metastatic outgrowth. Although switching from dormancy to active propagation sensitizes cancer cells to antimitotic drugs, the lack of effective timely monitoring strategies and the risk of resistance development deny the viewpoint of artificially and completely eradicating dormant cancer cells. 

Despite its significance in complementing tumor biology and enriching treatment strategies, the concept of dormancy remains vague and diverse. Based on their characteristics and biological functions, dormant cancer cells are also referred to as “drug persister cells”, “tumor-initiating cells”, “metastasis-initiating cells”, or “latency competent cells”, all of which cause confusion and hinder better understanding of dormant cancer cells. Several hallmarks, namely niche dependence, cell-cycle arrest, drug resistance, immune evasion, metastatic relapse potential, and reversibility to switch from dormancy to activation, have been proposed to help define and discriminate dormant cells. Of note, most of these hallmarks overlap those of CSCs, so occasionally “dormant cancer cells” and CSCs are considered to be the same. However, we need to address the existence of certain disparities between the two concepts and discuss their actual relationship in detail. Moreover, complex interactions between TIME and dormant cancer cells and CSCs warrant elucidation, as both of them could escape immunosurveillance from tumor-suppressive immune components, indicating their role in modulating local TIME into tumor-supportive facilitating cancer progression.

## 2. Stem Cell and Dormancy in Tumor Microenvironment

### 2.1. Relationship between CSC and Dormant Cancer Cell

Since the proposal of CSCs that explains the distinct differentiation status of cancer cells within the tumor mass, which contributes to intratumoral heterogeneity, the demonstration of CSCs in multiple hematological and solid malignancies, such as acute myeloid leukemia, breast cancer, glioma, and melanoma, have greatly revolutionized our knowledge of their biological essence and origin of their representative characteristics, including drug-persistence and enhanced tumor-initiating capacity. Various ATP-binding cassette (ABC) transporters, such as ABCG2, ABCC1, ABCB5, and P-gp, have been found to be overexpressed in BCSCs, all of which are involved in the transportation of chemotherapy drugs out of cancer cells and partially reveal one of the mechanisms for BCSC drug-persistence [19]. In addition to the contribution of CSC cell surface drug-resistance-related transporters, certain surface markers, including CD10, CD24, CD44, CD133, ALDH1, and GPR77, are employed for the identification and isolation of CSCs and have also been shown to be the cause of drug-resistance phenotype. The resistance of CSCs to drugs can also be modulated through certain components in the tumor microenvironment (TME). As observed in one study conducted by Su et al. [20], one group of cancer-associated fibroblasts, defined by a combination of CD10 and GPR77, participates in the formation of a pro-survival niche for breast cancer cells by sustaining cancer stemness, and thus contributing to chemoresistance. CSCs also exhibit enhanced potential and efficacy to generate neoplasms, as approximately 100 CD44+/CD24− BCSCs injected orthotopically could result in tumor formation in the breast [9].

As the concept of dormancy was first proposed more than half a century ago, the evolving understanding and knowledge about this distinguished cellular state has greatly prompted progress in exploring molecular mechanisms and triggering factors leading to drug resistance, local recurrence, and long-term distant metastasis. From the several abovementioned hallmarks that discriminate the dormant state, it is natural to notice that, indeed, there exist many similar properties between dormancy and stemness, such as treatment resistance and immunosurveillance evasion, serving as the origin and manifesting greater propensity for giving rise to metastatic relapse. Thus, the concept of dormancy and CSC is considered one and the same occasionally. However, there are essential differences between the two. First, as the origin contributing to the heterogeneous constituent of cancers, CSCs are also not identical, including their proliferation rate. Some CSCs are situated in a quiescent state with an extremely low self-propagation rate. This state, indeed, greatly resembles that of cancer-cell dormancy, while subgroups that will proliferate at a relatively rapid rate to compensate for the progeny pool also exist [21]. This dissection in their cell-cycling frequency depends on many factors, which may be attributed to their distinct differentiation status. Thus, since there is a broad range of CSCs, there is no direct and comprehensive evidence proving that CSCs have arrested cell cycle as the fundamental characteristic of dormancy. Furthermore, significant cell surface markers and transcription factors that distinguish CSCs, such as CD34, Nanog, SOX2, and Oct4, have only been detected in a small proportion of dormant cancer cells [22,23]. Most importantly, the proposal of the CSC complies with the hierarchy model of tumor composition, in which only a small proportion is thought to be tumorigenic and generate differentiated cancer cells, contrary to the stochastic model that each cell possesses an equal chance. Therefore, CSCs are located at the apex of the hierarchical structure. However, the dormant cells and their downstream activated proliferating cells manifest equal extent of differentiation, which ensures their reversible hallmark to switch between these two cell status. In summary, although many similarities exist between dormancy and CSC, they only share overlapping properties, and these two concepts should not be confused into one.

Despite their distinct phenotypes with regard to the definition of dormancy and the presence of specific stemness-related markers, CSCs and dormant cancer cells share several similar biological functions, indicating their potential comparability. Both CSCs and dormant cancer cells have the capacity to survive detrimental cytotoxic microenvironments through activated stress-tolerant signaling pathways and enhanced cellular autophagy, accounting for distant organ colonization in multiple carcinomas. In addition, the identical niche that dormant cancer cells occupy with CSCs highlights the similar extracellular cues that maintain their undistinguishable hallmarks [24]. As the most prominent metastatic site for breast cancer, detection of DTCs in the bone marrow serves as an independent prognostic factor and accounts for poor outcomes. Immunohistochemistry double/triple-staining of cytokeratin (CK), CD24, and CD44 was carried out by Balic et al. [25] to discriminate DTCs in the bone marrow and determine the presence and proportion of CD44+/CD24− BCSCs, thus evaluating metastatic potential. Among the CK+ DTCs, the mean proportion of stem/progenitor-like cells was observed to reach as much as 72%, higher than that of the primary tumor (<10%), which strongly indicates that CSCs comprise a major constituent of dormant DTCs within the bone marrow [26]. In addition, since CTCs transport via the bloodstream before arriving and colonizing the bone marrow, they tend to accumulate in sites that favor their enrichment and survival, among which the most important site is the perivascular niche (PVN) [27]. Previous studies have reported that occupation of PVN favors survival of hematopoietic stem cells (HSCs) and various tumor cells, including breast cancer DTC, as well as the regulatory role of integrin within PVN-mediating CSC stemness and DTC dormancy, resulting in immunosurveillance escape [28,29] and chemotherapy resistance [30]. Dormant DTCs in prostate cancer have been discovered to compete with HSCs for the PVN and osteoblastic niches, which would influence their seeding efficacy. Moreover, HSCs and breast cancer DTCs are trafficked to the bone marrow through the CXCR4-CXCL12 axis. Multiple cytokines, including transforming growth factor (TGF) beta2 (TGF-β2), growth arrest-specific 6 (GAS6), bone morphogenic protein 7 (BMP7), Wnt5α, and CXCL12, also correlate with the maintenance of dormant DTCs and CSCs generated from the bone marrow [31]. Heterogeneity was also observed among the stem-cell-like population within colorectal carcinoma, with some subgroups maintaining the progenitor pool and others responsible for developing into overt metastases [21]. In the dormancy-competent CSC (DCC) model proposed by Crea et al. [32], normal stem cells and CSCs situated at the early phase possess the capacity to switch from dormancy and proliferation (DCC) through reversible epigenetic modifications, which allow for neoplastic conversion and survival until irreversible genetic mutations occur. This leads to DCCs developing into more differentiated cells lacking dormancy-proliferation switching capacity. The DCC model is in accordance with the assumption that DTCs detached early from primary tumors may contain a larger fraction of stem-like cells with metastasis-initiating potential, as early DTCs with less chromosome gains or losses could seed distant metastases more efficiently.

In summary, although many similarities exist between CSCs and dormant cancer cells, they are still distinct concepts with essential differences. However, the partial comparability between them determines some common properties that maintain their biological characteristics, including common cell-intrinsic mechanisms and extracellular interactions with surrounding niches, which govern dormancy and stemness properties. The DCC model also bridges the relationship between CSCs and dormant cancer cells. Current well-recognized biomarkers of CSCs (Table 1) and factors leading to cancer-cell dormancy (Table 2) are summarized. Thus, elucidation of the similarity between CSC and dormant cancer cells may facilitate a better understanding of these two risk factors responsible for tumor initiation and cancer metastasis, thus aiding in the development of treatment regimens for inducing dormant subpopulations into more differentiated ones to inhibit activation of the harmless state and prevent lethal recurrence or metastasis.

### 2.2. Effect of Mesenchymal Stem Cells (MSCs) on Regulating Dormancy

Bone is the most prominent site for metastasis in multiple cancer types. Early phase occult DTC seeding in the bone and surviving the foreign environment contribute to their latency before converting into overt metastases, most of which remain in the dormant state and are also enriched for stem-cell-like subpopulations to escape elimination pressure from cytotoxic agents and immunosurveillance. Therefore, identifying and elucidating the signals contributing to DTC dormancy maintenance could be helpful for targeting strategy innovation, which may serve as the most convenient pathway to improve cancer patients’ prognosis and outcome. The dormant phenotype of DTC in the bone marrow mainly arises from the proliferation-restrictive microenvironment, apart from signals from several dormancy-inducing cytokines, including TGF-β2, BMP7, GAS6, leukemia inhibiting factor (LIF). Furthermore, existence of cellular compartments, such as MSCs, osteoblasts, and vascular endothelial cells, participate in the construction of this metastasis-inhibitory niche. MSCs and HSCs are two major normal stem cell populations residing in the bone marrow. MSCs demonstrated differentiation potency, migration capacity to tissue injury site and regeneration, immunomodulatory effect on regional TIME, and regulation of HSCs, which are mainly responsible for maintaining pluripotency of MSCs [81]. Thus, MSCs serve as one of the most important bone marrow resident-supporting stromal cells. Sufficient evidence revealed that DTCs share identical residing locations with HSCs. Strategies employed by HSCs to maintain their dormancy-like phenotype and proliferation-switch capacity may also be adapted by DTCs [82]. MSCs mainly interact intercellularly with DTCs through three mechanisms: transportation of cellular contents through gap-junction intercellular communication (GJIC), microvesicles mainly comprising exosomes, and release of growth-suppressive cytokines in a paracrine manner.

A study conducted by Lim et al. [83] demonstrated the dominant role of GJIC in mediating cellular non-coding genetic material transportation to arrest breast cancer proliferation. The authors co-cultured T47D and MDA-MB-231 cell lines with bone-marrow stromal cells, and an increased proportion in the G0/G1 phase was detected, accompanied by decreased cyclin D1 and CDK4-mediated G1 to S-phase transition. Detailed mechanistic insight indicated that GJIC between breast cancer cells and MSCs facilitated the intercellular transfer of several miRNAs, including miR-127, miR-222, and miR-223. The miRNAs participated in the downregulation of CXCL12, which is ubiquitously expressed in the bone marrow and is responsible for chemotactic migration of cancer cells from the primary site toward the bone. Attenuation of CXCL12 significantly reduced cellular proliferation after accumulation of the three miRNAs as during the co-culture.

In addition, the interaction between MSCs and DTCs to maintain their dormant phenotype is mainly achieved via the abundant release of exosomes. Being recognized as a subset of extracellular vesicles originating from endosomes and containing numerous cellular contents, including nucleic acids, proteins, lipids, amino acids, and metabolites, exosomes and their biological functions have evolved from simply releasing unnecessary cellular constituents to regulating intercellular communication [84]. Initially, a breast-cancer-cell-line BM2 was generated through in vivo clonal selection with enhanced osteo-metastatic capacity and co-cultured with bone-marrow-derived MSCs (BM-MSCs), which suppressed the proliferation of BM2 cells and attenuated invasion capacity and docetaxel resistance property. Acquisition of this dormancy phenotype after culturing BM2 cells with purified exosomes secreted from BM-MSCs prompted screening of miRNAs enriched in exosomes, accounting for the dormant state induction. Among them, high expression of miR-23b was associated with a reduced proliferation rate by targeting downstream MARCKS, which results in attenuated cell cycling and motility [85]. Furthermore, miR-222 and miR-223 were also found to be involved in dormancy induction in this manner, promoting dormancy of DTCs and conferring them with drug-resistant characteristics. Moreover, antagomiR-222/-223 embedded in MSCs was administered in vivo to target the dormant subset and reverse the inferior outcome. The results showed promising findings, as antagonism of miR-222/-223 sensitized the dormant DTCs to cisplatin-based chemotherapy and improved host survival, strongly highlighting the crucial role of miRNA-containing exosomes in mediating the dormancy-inducing function of MSCs on DTCs, as well as the targetable and reversibility of this process [86]. More recently, a stepwise modification of MSCs to DTCs into dormant state was proposed, in which Wnt/β-catenin regulation mediates early metastasized breast cancer cells de-differentiating into preliminary CSCs at the bone marrow perivascular niche. Furthermore, CSC-phenotypic cells interact with MSC-secreted extracellular vesicles, including exosomes, to initiate transition into cycling quiescence and activation of DNA repair, thus de-differentiating into a more complete CSC subpopulation. This mechanism elucidated the precise process of dormancy induction within the perivascular niche and under BM-MSC-secreting exosome induction [87].

**Figure 1:**
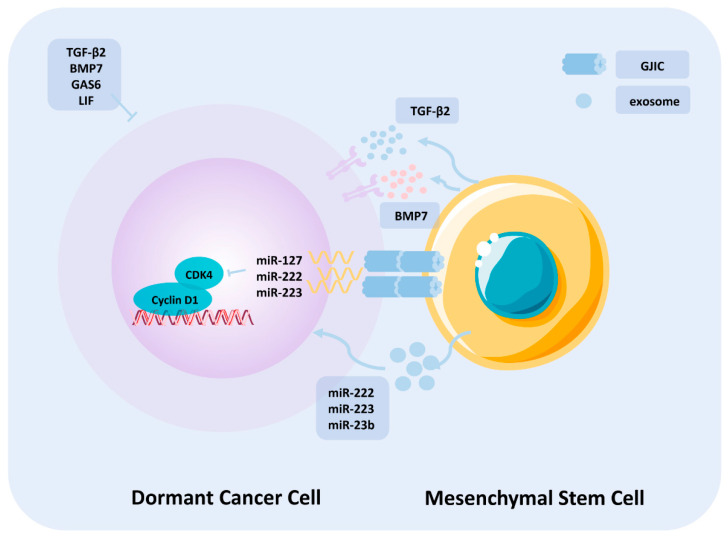
Mechanism of mesenchymal stem cells maintaining cancer cells’ dormancy. Apart from the cytokines present in the tumor microenvironment responsible for the maintenance of cancer-cell dormancy, MSCs also exert pivotal functions in arresting cell-cycle progression, mainly through three mechanisms: (1) intercellular communication of miRNA directly through gap-junction intercellular communication (GJIC) to reduce transcription of cell-cycle components, thus blocking the G0/G1 transition; (2) transportation of miRNA-containing exosomes to exert the same effect as direct communication; and (3) secretion of TGF-β2 and BMP7 from NG+/Nestin+ mesenchymal stem cells to activate p27 and p38 to induce dormancy. Abbreviations: BMP7, bone morphogenetic protein 7; GAS6, growth arrest specific 6; LIF, leukemia inhibitory factor; GJIC, gap-junction intercellular communication.

In addition, specific subgroups of MSCs have also been identified and validated for their ability to induce DTC dormancy via secretion of proliferation-limiting cytokines and release through ligand-receptor combination. Nobre et al. [88] demonstrated one distinct NG+/Nestin+ MSC in the bone marrow and its abundance resulted in intensified secretion of TGF-β2 and BMP7 and activation of common downstream p27, p38, and SMAD through binding with TGFBRIII and BMPRII, respectively, inducing cascade accounting for breast cancer latency in bone marrow. Furthermore, genetic knockout of the NG+/Nestin+ MSC resulted in accelerated metastatic outgrowth, and clinical data analysis based on biopsies from hormone receptor-positive/HER2-negative breast cancers revealed that patients without long-term relapse or bone metastases exhibited a higher frequency detection of TGF-β2 and BMP7 in the bone marrow, suggesting that NG+/Nestin+ MSCs are crucial in the dormancy and proliferation homeostasis maintenance of bone-marrow-residing DTCs [88]. Taken together, MSCs within the TME exert potent dormancy induction and maintenance capacity through intercellular communication via multiple miRNAs and cytokines (Figure 1). Further investigations are warranted to elucidate the mechanism underlying the development of targeted strategies against the dormant subpopulation tamed by MSCs.

## 3. Immune Component in Regulating CSCs and Dormancy Cancer Cells

Solid tumor masses are composed of parenchymal cancer cells, as well as multiple types of stromal cells and soluble cytokines constituting the TME, which play an indispensable role in supporting cancer progression. Based on the progressive knowledge about TME, concentrating solely on cancer cells may be insufficient to manage malignancies. Numerous strategies targeting TME have been developed and have achieved considerable treatment efficacy, such as anti-angiogenesis therapy and harnessing effective immune components for elimination of neoplasms, mainly through blockage of immune checkpoints, including PD-1, PD-L1, and CTLA4. An effective tumor-suppressive immune niche has been shown to correlate with improved benefits from both neoadjuvant and adjuvant chemotherapy, highlighting the vital role of the TIME in the complete elimination of cancer cells. However, the complex interaction between cancer cells and host immunity contributes to the development of immunoediting, which consists of three phases: tumor elimination, equilibrium, and tumor escape as an antitumor inflammation switch from acute to chronic immunity. Initially, recognition of the non-self-component triggers activation of innate immunity and dendritic cells’ processing and presenting neoantigens to prime tumor-specific T cells against cancer cells. Ultimately, after enduring the equilibrium phase where proliferation and apoptosis reach balance, a shift from acute to chronic inflammation modulates TIME into tumor-permissive, including immune cells (regulatory T cells (Treg), myeloid-derived suppressor cells (MDSC), and tumor-associated macrophages (TAM)) and stroma (fibroblast and endothelial cells). The process of immunoediting is accompanied by upregulation of immune checkpoint expression and activation of immune-suppressive metabolic pathways in TIME, which is partly contributed by the regulation of cancer cells, especially the CSC subset. In addition, systemic and regional inflammation, together with immunity alteration, account for the awakening of dormant DTCs. This leads to metastatic outgrowth based on the observation that surgical removal of the primary tumor may trigger DTCs into metastatic outgrowth, and pre-operative administration of anti-inflammatory drugs reduces distant recurrence rate within 18 months after breast cancer surgery [89]. Thus, elucidating the mechanism of CSC modulating TIME into tumor-permissive, as well as the process by which immune cells cause DTCs to exit from dormancy, will serve as strategies against these refractory cancer-cell subsets. Here, we review the present reports on the interaction between CSCs and several kinds of immune cells, along with their influence on cellular dormancy.

### 3.1. Tumor-Associated Macrophages

Macrophages belong to myeloid cells and play a major role in anti-infection and tissue homeostasis maintenance by directly engulfing foreign material and tumor cells, mediating innate immunity, and facilitating execution of specific adaptive immunity [90,91]. Depending on the cytokines, chemokines, and tumor-favoring microenvironments in tumor mass, monocytes in peripheral circulation can be recruited to the TME and polarize into TAMs [92]. Macrophages originate from prenatally developed and differentiated tissue-resident macrophages (TRM) localized to various tissues and organs during embryonic development, as well as those originating from peripheral circulating precursor monocytes and constitute the major proportion of macrophages [93]. TRMs have been reported to be involved in the constitution of the niche supporting several kinds of stem cells, thus leveraging a similar mechanism for the maintenance of CSCs [94]. Heterogeneity also exists across macrophages, and based on their distinct functions, they can be categorized into pro-inflammatory M1 macrophages and immunosuppressive M2 macrophages [95]. Several pro-inflammatory cytokines, including interferon (IFN)-γ and GM-CSF, are produced by M1 macrophages and play an important role in defense against foreign pathogens [96]. Meanwhile, M2 macrophages participate in several biological processes, such as tissue integrity maintenance, allergic reactions, and angiogenesis [73]. However, this classification is somewhat abstract with other macrophage subsets with distinct properties, such as CD169+ and TCR+ macrophages [95], although it has not been thoroughly clarified. TAMs consist of a spectrum of macrophages with various activation states, with most properties matching those of M2 macrophages. Thus, TAMs are sometimes defined as M2 macrophages in a narrow sense, although further exploration revealed that TAMs share both M1 and M2 molecular signatures.

**Figure 2:**
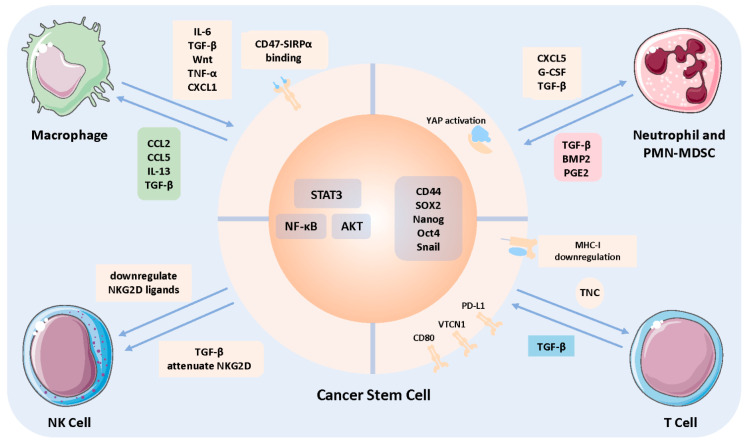
Interaction between cancer stem cells and immune components. Interaction between CSCs and immune cells are mutual and complex, and their interaction are accomplished mainly through intercellular cytokines communication. (1) Interaction with macrophage: CSC-secreted CCL2, CCL5, and TGF-β lead to immune incompetency of macrophage, thus preventing eradication; macrophage-secreted IL-6, Wnt, and TGF-β could enhance CSC stemness; moreover, the CD47 “do not eat me” signal presented by CSC avoids phagocytosis. (2) Interaction with TAN and PMN-MDSC: TAN-secreted TGF-β and BMP2 lead to dedifferentiation of cancer cells into CSC, and PMN-MDSC could upregulate stemness regulatory transcription factors; CSC-secreted chemokines recruit more TAN and PMN-MDSC to form the suitable immune microenvironment. (3) Interaction with NK cell: Owing to the prominent cytotoxic function of NK cells, CSCs mainly downregulate NKG2D ligands level to avoid killing. (4) Interaction with T cell: CSCs attenuate MHC-I expression and upregulate immune checkpoints to prevent immune recognition and attack, conversely, CSC-secreted tenascin C retards T cells proliferation to thrive. Overall, CSC-secreted cytokines are responsible for inducing incompetency of immune cells; and immune-cell-released ones are crucial for maintenance and strengthening of stemness to enhance the refractoriness of cancer. Abbreviations: BMP2, bone morphogenetic protein 2; G-CSF, granulocyte colony stimulating factor 6; PGE2, prostaglandin E2; SIRPα, signal regulatory protein alpha; TNC, Tenascin C; VTCN1, V-set domain-containing T-cell activation inhibitor 1; YAP, Yes1-associated transcriptional regulator.

In a study conducted by Weinberg et al. [97], CD68+ macrophages were demonstrated to be localized adjacent to CD90+ cancer cells, which possess several characteristics of CSCs. These findings firmly implicate the close interaction between TAMs and CSCs. The available in vitro results demonstrating the bidirectional crosstalk between TAM and CSC are virtually based on their co-culture with the conditioned medium of the other. For example, co-culture of various types of cancer cells, including HCC and glioblastoma with CSC-conditioned media, promoted the release of numerous chemokines and cytokines favoring tumorigenic macrophage factors [98,99,100,101], including CCL2, CCL5, CSF1, GDF-15, IL-13, TGF-β, and WISP1 (Figure 2). A further process of macrophages with these cytokines manifests an immune incompetency phenotype, implicating CSCs could influence the polarization of macrophages and modulate them into tumor-permissive. Additionally, adding conditioned medium of M1 macrophages to luminal type breast cancer cells intensified the CSC-related phenotypes and enhanced epithelial–mesenchymal transition (EMT) properties. Further mechanistic insight revealed that TAM could also influence CSC phenotypes through secretion of several soluble cytokines, including IL-6, TGF-β, and Wnt, further increasing CSC self-renewal and tumor-initiating capacity through activation of downstream NF-κB, STAT3, and Akt signaling molecules (Figure 2) [50]. Moreover, upon administration of conditioned medium from TAM, several breast cancer cells manifested increased stemness and EMT-related activity. In MCF-7 cells, this was achieved by TNF-α-mediated stabilization of Snail [102]. CXCL-1 secreted by TAMs could enhance SOX4 expression via NF-κB activation in a MMTV-PyMT tumor model [103]. Based on the above findings, the interaction between TAM and CSCs is mutual and complex. Aside from polarization modulation effect of CSCs on macrophages, CSCs could also adapt an anti-phagocytosis strategy through upregulating cell surface CD47 expression and bind with signal regulatory protein alpha (SIRPα) on macrophages to phosphorylate the ITIM motif, conveying the “do not eat me” signal to escape elimination pressure from tumor-suppressive macrophages (Figure 2) [104]. Preclinical data on blockage of the CD47-SIRPα axis demonstrated enhanced phagocytosis of tumor cells in multiple types of cancer, which provides promising translational value for targeting interactions between TAMs and CSCs.

**Figure 3:**
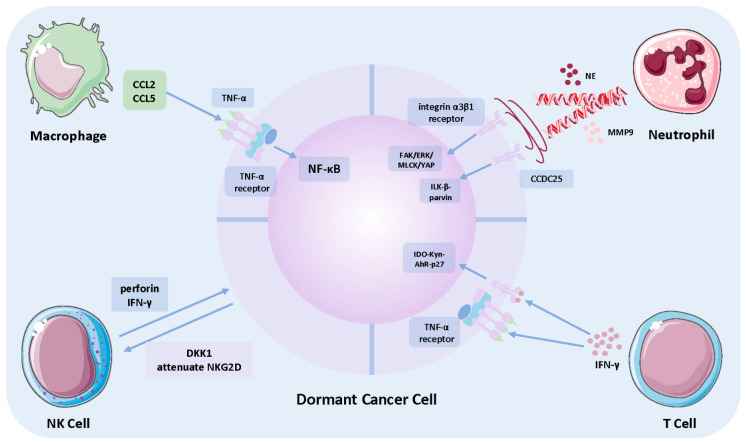
Crosstalk between dormant cancer cells and immune components. The mechanism and effect of tumor-associated macrophage, tumor-associated neutrophil, NK cells, and T cells on dormancy varies. (1) Crosstalk with Macrophage: CCL2 and CCL5 in TME recruit macrophages and polarize them into tumor-permissive M2 TAM, leading to dormancy awakening via upon TNF-α/NF-κB axis. (2) Crosstalk with TAN: TANs have been revealed to release neutrophil extracellular trap to activate dormant cancer cells into proliferation state either through integrin α3β1 receptor or CCDC25. (3) Crosstalk with NK cell: NK cell-secreted perforin and IFN-γ restrain cancer cells in dormant state; DKK1-expressing dormant cancer cells adopt similar strategies as CSCs to attenuate NKG2D expression to escape killing. (4) Crosstalk with T cell: T-cell-mediated dormancy induction is predominantly achieved via IFN-γ secretion and the downstream IDO-Kyn-AhR-p27 axis. Abbreviations: CCDC25, coiled-coil domain containing 25; DKK1, dickkopf WNT signaling pathway inhibitor 1; ERK, extracellular signal-regulated kinase; FAK, focal adhesion kinase; MLCK, myosin light-chain kinase; YAP, Yes1-associated transcriptional regulator; ILK, integrin linked kinase; IDO, indoleamine 2,3-dioxygenase; Kyn, kynurenines; AhR, aryl hydrocarbon receptors; NE, neutrophil elastin; MMP9, matrix metalloproteinase-9.

Metastatic dormancy has been established as the conceptual basis for tumor relapse many years after complete control of the tumor, while within the same tumor type, a small proportion of patients develop overt metastases soon after resection of the primary tumor compared with those surviving with long-term recurrence-free tumors. These observations prompted researchers to explore tumor-extrinsic factors associated with triggering of dormant cell metastatic outgrowth. As demonstrated by a previous study, systemic inflammation and regional immune component alteration contribute to awakening of the dormant cancer cells as perioperative administration of anti-inflammatory drug significantly attenuate relapse risk after tumor resection [105,106]. To explore precisely the exact executor responsible for this awakening role, analysis on peripheral blood leukocytes and cytokines were analyzed in mice undergoing surgical wound recovery and control, and the results revealed that circulating monocytes and neutrophils, along with IL-6, G-CSF, and CCL2, were elevated significantly [106]. The negative correlation between CD11+ myeloid cells and CD8+ T cells, and the positive correlation with cancer cells strongly indicate that TAMs could reduce T-cell dormancy maintenance function, and their elevation after inflammation is associated with an increased risk of metastatic outgrowth. CCL2 knockout, which is pivotal in attracting and promoting differentiation of peripheral monocytes into TAM, significantly reduced tumor growth [106]. These results indicate that TAM could induce dormant cells to outgrow by interrupting tumor-suppressive TIME. In addition, another study has revealed the role of CCL5 in depositing TAM, leading to metastasis [107]. Moreover, HER2-downregulation contributed to the expression pattern of pro-inflammatory signature leading to elevation of several cytokines and chemokines, including CCL5. This was completed by the activation of TNF-α/NF-κB axis and results in increased monocyte recruitment and TAM differentiation, leading to activation of the dormant subset (Figure 3). Thus, targeting the TNF-α/CCL5/macrophage axis may serve as an optimal strategy for the prevention of long-term metastasis.

### 3.2. Tumor-Associated Neutrophils and Polymorphonuclear MDSC (PMN-MDSC)

Neutrophils constitute a substantial proportion of leukocytes serving as the first-line defense against invading foreign pathogens and responding to tissue damage [108,109]. Recruitment of neutrophils to sites of acute inflammation exerts a direct cytotoxic effect through the secretion of multiple enzymes, mainly myeloperoxidase (MPO) [110], neutrophil elastase [111], and matrix metalloproteinases [112], which play a pivotal role in the early acute phase of inflammation. Clearance of neutrophils by apoptosis or engulfment from macrophages avoids regional inflammation switching into chronic inflammation causing tissue damage [113]. Aberrant accumulation of neutrophils within tissues results in sustained inflammation, which eventually contributes to tumorigenesis [114], highlighting the multifaceted role of neutrophils in the TME. Similar to TAM, neutrophils within tumors exhibit high plasticity and can switch phenotypes depending on regulatory cues from the surrounding environment, and are mainly classified into N1- and N2-tumor-associated neutrophils (TAN) based on their functional differences owing to the lack of distinguishable markers [115,116]. TGF-β secreted by tumor cells could induce neutrophils into N2 TAN, manifesting a tumor-permissive phenotype that mediates immunosuppression, angiogenesis, and metastasis [115]. Meanwhile, depletion of TGF-β or administration of type 1 IFNs could polarize neutrophils into N1 TAN, exhibiting antitumor phenotype [117]. Apart from N1 and N2 TANs, one subset of MDSCs and PMN-MDSCs have been identified to share the same origin and identical differentiation portraits with neutrophils to further complement the spectrum of these heterogeneous populations [118]. Although phenotypes similar to neutrophils manifest CD11b+/Ly6G+/Ly6C^low^ in mice and CD14−/CD11b+/CD15+ (CD66b+) in humans [119], functional annotations and transcriptomic analysis have defined their distinctions: TANs exhibit tumor-promoting roles by exerting innate immune inflammation; CCL2 and CCL17 secreted by TANs recruit peripheral monocytes to further differentiate into TAMs and Tregs to suppress effective antitumor immunity, as described above [120,121], and PMN-MDSCs promote tumor progression by suppressing adaptive immune response via inhibiting T-cell function [122]. Transcriptomic analysis also revealed elevated endoplasmic reticulum stress response and lectin-type oxidized LDL receptor 1 expression in PMN-MDSC [123], which would help distinguish these cells from neutrophils within peripheral blood and tumor tissues across multiple solid cancers. Recent studies have revealed that reverse migration from the inflammatory site reverts blood circulation, providing an additional pathway for neutrophil clearance [124]. A recent study captured neutrophils in hepatic sterile inflammatory sites moving reversely into peripheral circulation and seeding into the lung and bone marrow [113]. Considering that the bone and lung serve as principal sites for cancer metastasis, neutrophils are thought to be involved in the construction of a pre-metastatic niche that facilitates DTC seeding, and mutual crosstalk could facilitate tumor progression. Here, we review the present findings on the interaction between CSCs and TANs, as well as PMN-MDSCs, and outline the role of neutrophils in awakening dormant DTCs.

Aside from the complex regulatory mechanism of TANs and PMN-MDSCs in modulating tumor-permissive TIME facilitating initiation and progression, they have also recently been found to be involved in CSC stemness across several cancers, and CSCs could reverse their infiltration. TANs secreting TGF-β and BMP2 were found to be involved in the dedifferentiation of HCC cells into the CSC phenotype through upregulation of the NF-κB signaling pathway [115,125], and HCC-derived CXCL5 was responsible for chemotactic recruitment of TANs to increase infiltration (Figure 2) [125]. In the context of PMN-MDSCs, co-culture of PMN-MDSCs with multiple myeloma cells upregulated key stemness-related transcription factors, including Nanog, OCT4, and SOX2 (Figure 2) [126]; prostaglandin E2 (PGE2) secretion from PMN-MDSCs was found to induce expansion of the ALDH+ CSC subset in human cervical cancer [127], and PMN-MDSCs upregulate CD44 and CD133 expression levels through phosphorylation of STAT3 in colorectal carcinoma [128]. G-CSF overexpression in cervical cancer could generate more PMN-MDSC and ALDH+ CSCs compared with control; YAP activation in prostate cancer is responsible for CXCL5 upregulation to attract CXCR2+ PMN-MDSC [129], and TGF-β secretion by CD133+ melanoma cells could increase surrounding PMN-MDSCs and TAM infiltration (Figure 2) [130]. Thus, TANs and PMN-MDSCs could cooperate with CSCs to enhance their stemness features and further modulate tumor-permissive TIME.

Metastatic dormancy refers to the viable but undetectable situation of DTCs until certain awakening signals arise during the proliferation phase. Since systemic and sustained inflammation correlates with shortened metastasis-free survival and neutrophils comprise the major executor in immune inflammation, it is reasonable that neutrophils account mainly for awakening dormant DTCs. Neutrophil extracellular traps (NETs) were first discovered by Brinkmann et al. [131] as the major pathway for neutrophil defense against bacterial infection. NET has been found to have a non-negligible role in promoting cancer metastasis. NET is an extracellular web-like structure composed of chromatin DNA filaments, histones, and antimicrobial enzymes, such as NE, MPO, and MMP9, to trap microorganisms and sequester CTCs in the context of its tumor promotion property [132]. Citrullinated histone H3 could serve as a biomarker for detecting NET [133], whose elevation in peripheral blood and vital organs has been revealed to correlate positively with distant metastasis risk. Albrengues et al. [134] found that sustained inflammation stimulated the formation of NETs, while NE and MMP9 exert their degradation function on extracellular laminin. Furthermore, the proteolytically remodeled laminin residues transformed into the integrin α3β1-activating epitope, initiating downstream FAK/ERK/MLCK/YAP signaling pathway to awaken dormant DTCs and contribute to lung metastasis outgrowth (Figure 3) [134]. In addition to the role of NET-composed enzymes in triggering dormancy awakening, the DNA component of NETs (NET-DNA) has more recently been characterized for their chemo-attractive role in chemotactic migration of DTCs to sites of future metastasis occurrence [135]. In their study, elevated NET levels in the liver could predict future liver metastasis and mediate detached DTCs migrating to the liver rather than other organs; therefore, NET-DNA was identified as an active attractive factor rather than passively waiting for trapping DTCs. In addition, CCDC25 was identified as a transmembrane protein in cancer cells for sensing distant NET-DNA signals, and activation of the ILK-β-parvin pathway downstream of CCDC25 enhanced breast-cancer-cell motility towards NET (Figure 3). Thus, inhibition of CCDC25 could effectively prevent dormancy subpopulation awakening and reduce metastasis-related mortality.

### 3.3. Natural Killer (NK) Cells

NK cells are a subpopulation of cytotoxic lymphocytes belonging to the innate immune system [136,137], and they play a pivotal role in the first-line defense against virally infected and neo-transformed cells mainly via cytotoxic effects and the release of pro-inflammatory cytokines [138]. NK cells are the first identified subtype of innate lymphoid cells (ILCs), and together with ILC1, ILC2, and ILC3, they originate from the same common lymphoid progenitor cells as B cells and T cells [139,140]. Originally, CD34+/CD45RA+ hematopoietic progenitor cells migrate from the bone marrow to various destinations and differentiate into mature NK cells characterized by CD3−/CD56+ mainly induced by IL-15 [141,142]. In contrast to B cells or T cells, which recognize foreign antigens through somatically arranged antigen-specific receptors, NK cells exert their function mainly through several activating and inhibitory receptors, and the balance between these receptors determines the cytotoxic response or immune repression [143]. The low-affinity IgG Fc region receptor, also known as CD16, is the most potent activating receptor that crosslinks with the Fc region of the IgG antibody labeled on target cells and mediates the antibody-dependent cell-mediated cytotoxicity (ADCC) effect [144]. In addition to CD16, the natural cytotoxicity receptor family, including Nkp30, Nkp40, Nkp44, and Nkp46, could directly bind with virally infected or tumor-associated epitopes and activate downstream cascades to mediate cytokine production and cytotoxicity [145]. NKG2D and NKG2C are also important activating receptors, and MHC class I polypeptide-related sequence (MIC) A, MICB, and retinoic acid early transcript/U16 binding protein are typically expressed on multiple abnormal cells and serve as ligands for NKG2D [146]. NK cells have been originally designated for their considerable capacity of killing tumor cells, and the process and mechanism mediating their effect mainly includes ADCC and “missing-self” regulation; the latter one refers to the process that NK cells are normally repressed via binding with MHC I molecules [147], while cancer cells’ evolution trend towards downregulating their MHC I expression could reactivate NK cells to exert their effector function and supplement the immune evasion from CD8+ T cells [148]. While NK cells consistently defend against tumor cells, immunosuppressive strategies have been adopted by tumor cells to evade slaughter from NK cells, mainly through secretion of multiple cancer-associated soluble immunosuppressive molecules, including IL-10, indoleamine 2,3-dioxygenase, PGE2, and TGF-β) into the TME [149,150,151]. Among these cytokines, TGF-β is the most abundant and is secreted not only from cancer cells, but also from suppressive immune components, including Tregs, MDSCs, and TAM [151], and functions as the most potent regulator mediating cancer-cell immunosurveillance evasion.

Crosstalk between CSCs and NK cells still has a limited understanding, except for several mechanisms discovered for CSCs to escape from NK cells. CSC subsets in multiple cancers, including those in lung cancer, colorectal carcinoma, melanoma, glioblastoma, and leukemia [152,153,154,155], have been discovered to attenuate the expression of NKG2D ligand, which would abrogate the capacity to recognize and initiate effective cytotoxicity. In addition, CD133+ glioblastoma CSCs have also been discovered to secrete TGF-β, which directly downregulates the NKG2D expression level and facilitates avoidance of cellular lysis (Figure 2) [156,157]. The negative strategies employed by multiple CSCs prompt further discovery and enrichment of mechanisms for the performance of the full degree cancer-limiting effect of NK cells.

NK cells play a significant role in maintaining both tumor-mass and cancer cellular dormancy. Wu et al. [158] have a significantly higher percentage of NK cells was detected in mice with dormant tumor mass compared with those with progressing sarcoma, suggesting that NK cells are pivotal in limiting the proliferation of cancer cells and reach equilibrium with apoptosis to maintain tumor mass dormancy. Cytokines secreted by NK cells into the TME seems to play the major role in restraining cancer cells at the dormant state. Brodbeck et al. [159] verified that NK cells are indispensable in restraining tumor growth at both primary site and the metastatic lesion in colon cancer. Through further computation analysis they observed that the perforin-mediated cytotoxicity of NK cells serve as the main force leading to cancer cells remaining dormant [159]. Moreover, a recent study provided direct evidence that abundance of NK cells reservoir pool determines entering into or exiting from dormant state in metastatic breast cancer cells to liver. Sufficient stimulation from IL-15 could ensure abundant amount of NK cells surrounding the metastasized cancer cells in the TME, which would subsequently secrete IFN-γ to restrain cancer cells in dormant state (Figure 3). Moreover, under a condition such as liver damage, activated hepatic stellate cells could secrete CXCL12 to bind with NK cells’ surface CXCR4 and result in a quiescent phenotype of NK cells, and the incompetent NK cells eventually lose their dominance and result in re-population of the metastasized cancer cells [160]. Apart from the mechanism of NK cells maintaining dormancy, several kinds of cancers adopt strategies to evade. For instance, multiple dormant cancer cells express dickkopf-related protein 1, which is one inhibitor of Wnt and could lead to downregulation of ULBP level in NK cells [161], attenuated crosslinking between ULBP and NKG2D results in abrogated NK cells effect (Figure 3). Taken together, the prominent effect of NK cells on diminishing the dormant subpopulation has brought great promise for the development of NK cell-directed immunotherapy for the purpose of completely eradicating refractory dormant cancer cells.

### 3.4. Effector T and Tregs

T cells constitute the major proportion of lymphocytes intratumorally and represent the most heterogeneous subset with varying and complex functions [162], and the most concentrated subsets include the tumor-repressive effector T cells, including CD4+ T cells and CD8+ T cells, and tumor-permissive regulatory T cells, mostly referring to Tregs. CD8+ T cells are also called cytotoxic lymphocytes (CTLs), which exert potent antitumor effects by recognizing neoantigens expressed by cancer cells or presented by antigen-presenting cells, subsequently initiating cytokine secretion or releasing granzyme B and perforin to mediate targeted cancer-cell eradication [163]. Aside from CD8+ T cells, CD4+ T cells, also known as T-helper (Th) cells, display a non-negligible role in defense against tumor as well, and the mechanisms behind them are diverse. First, Th cells could facilitate CD8+ T cells to exert a cytolytic effect, which could directly damage cancer cells [164]. Second, TME modulation through tumor-limiting cytokine release could hinder the development and progression of tumors [165]. More importantly, Th cells facilitation to CD8+ T cells ensures avoidance of negative regulation on the most competent subpopulation, thus enhancing antitumor response [166]. Meanwhile, Tregs are normally characterized by CD4+/CD25+/Foxp3+ and constitutively express CTLA4 to competitively bind to CD80/CD86 against CD28 [167,168]. The abundance of Tregs in TIME has been shown to correlate with immune suppression and negative outcomes in multiple cancers [169,170,171,172]. Their tumor-permissive effect is achieved either by restraining the proliferation of tumor-specific effector cells or by limiting the secretion of IL-2 and IFN-γ owing to the expression of CTLA-4, glucocorticoid-induced tumor necrosis factor receptor (GITR), and Foxp3 [173,174,175]. Attempts aimed at potentiating the cytotoxic effect of effector T cells or repressing Treg activity, including immune checkpoint inhibitors, have been developed and applied to revolutionize cancer therapy, which has achieved considerable benefits. However, a low fraction of treatment efficiency indicates that resistance mechanisms are still underway, especially the crosstalk between T cells, CSCs, and dormant cancer cells.

Based on preclinical findings on melanoma and B-cell lymphoma, development of local recurrence or overt metastases is postponed due to the presence of dormant DTCs, and the duration of the latency period relies on the content of effector T cells [176,177]. Depletion of CD8+ T cells shortens the time for re-emergence of overt foci, and this phenomenon is mainly attributed to decreased secretion of IFN-γ. In accordance with the above finding in NK cell-mediated liver metastasized breast-cancer-cell dormancy, IFN-γ is responsible for inducing G0/G1 phase arrest [178], as well as maintaining a low proliferation rate of the tumor-repopulating cells in hepatic carcinoma and melanoma [179], which is achieved through the IDO1-kynurenine-aryl hydrocarbon receptor-p27 axis (Figure 3) [180]. Apart from the dominant effect of CD8+ T cells in enforcing dormancy, CD4+ T cells secrete IFN-γ, and the binding TNFR1 can induce tumor growth arrest and establish tumor dormancy in a mouse model of pancreatic cancer [181]. An increased CD8+ /CD4+ T cell ratio has also been discovered in dormant tumors compared with developing ones, which further suggests the dominant force exerted by CD8+ T cells [158]. The crosstalk between CSCs and effector T cells eventually contributes to evasion of CSCs from T-cell-mediated cytotoxicity or attenuation of the anti-tumorigenic capacity of T cells, including downregulation of CSC surface MHC-I expression to prevent immune recognition and upregulate CD80 expression in a TGF-β-dependent manner to induce immune tolerance (Figure 2) [154,182]. The latter highlights the strategy employed by CSCs to enrich inhibitory immune checkpoint ligands, including PD-L1 and VTCN1 (Figure 2) [183,184,185]. In addition, tenascin C containing extracellular vesicles could interfere with the α5β1 integrin receptor on T cells to impair cellular proliferation through downregulation of AKT and ERK phosphorylation (Figure 2) [186].

Direct evidence demonstrating the role of Tregs in regulating tumor dormancy is limited and controversial, and the content of Tregs is lower in dormant sarcomas than in progressing sarcomas [158], thus corresponding with the immunosuppressive role that Tregs exert. However, the presence of dormant cancer cells in B-cell lymphoma is accompanied by elevated Treg levels [187], and this suggests that the regulation of dormancy imposed by Tregs is tumor-type specific and warrants further investigation. In the context of CSCs, the interaction between CSCs and Tregs is mainly achieved through the secretion of chemokines and cytokines to facilitate recruitment of Tregs into TIME. CCL1, CCL2, and CCL5 are the main chemokines released by CSCs. CCL1 mediates the migration of Tregs to SOX2+ breast cancer cells [188], and CCL2–CCR4 and CCL5CCR5 binding are responsible for the attraction of Tregs in glioblastoma and ovarian cancer, respectively [189,190]. In addition, elevated expression of IDO1 and TGF-β originating from CSCs also accounts for the recruitment of Tregs [191,192]. Thus, the above findings regarding the crosstalk between T cells, dormant cancer cells, and CSCs are centered around strategies to evade immunosurveillance or attenuate cytotoxic capacity. The underlying mechanism still warrants further investigation and enrichment to guide effective target strategies to overcome the immunosuppressive effect and control this detrimental cancer-cell subpopulation.

## 4. Discussion

CSC and dormant subpopulations within primary lesions or metastatic sites account for several detrimental properties of malignancies, leading to treatment failure and eventually to the most devastating outcome. Although they are functionally and phenotypically similar, and sometimes confused into one concept, CSCs and dormant cancer cells can hardly be mixed into one. As discussed above, CSCs and dormant cancer cells share overlapping characteristics, yet essential distinctions exist between them, thus highlighting that heterogeneity is also present in the CSC population, both in the dormant and relative proliferative state. However, the resistance of CSCs to chemotherapy and DTCs in a dormant state evade pressure from genotoxic agents, radiotherapy, and other biological interventions. This calls for urgent development of effective strategies to eradicate them, preventing occult lesions from developing into occult metastases. The striking similarities shared between CSCs and dormant cancer cells will provide novel co-targets in the near future. In addition, the interaction between dormant cells and MSCs, the most prominent stem cells, within the most commonly occurring metastatic site in the bone enrich numerous dormancy-inducing signals, and this enrichment occurs mainly through intercellular communication mediated by miRNA-containing exosomes and direct regulation of TGF-β and BMP7 in a paracrine manner. The dormancy-regulating capacity of MSCs further unravel the “stemness force” in balancing local tumor initiation and progression. As the most complex and crucial factor in modulating CSC and dormancy, the immune components have been widely reported for their dual regulatory role in the crosstalk between local TIME and CSCs and dormant cancer cells. Thus, the underlying mechanism should be elucidated and summarized. This would be of great significance in enriching the knowledge on the eradication of CSCs and reverse modulation of TIME into a tumor-suppressive one under the delicate mechanisms exerted by CSCs, as well as identifying the potential awakener leading to tumor re-initiation.

This review sheds light on analogues between cancer stemness and dormancy, and further research is warranted for the development of effective therapeutic avenues through several shared targets, which may potentially “achieve two aims at once” in the near future. In addition, this review is the first to list the immune components that have been reported to exert simultaneous regulation of CSCs and dormancy; thus, it may inspire future strategies for overcoming these tumor promoters.

## Figures and Tables

**Table 1:** Typical biomarkers of cancer stem cells in solid tumors.

Markers	Tumor Type	Reference
**Surface Markers**		
CD24	breast cancer, gastric cancer, liver cancer, and colorectal cancer	[33,34,35,36]
CD44	lung cancer, breast cancer, gastric cancer, liver cancer, and colorectal cancer	[37,38,39,40,41,42]
CD90	lung cancer, breast cancer, gastric cancer, and liver cancer	[43,44,45,46]
CD133	lung cancer, breast cancer, gastric cancer, liver cancer, and colorectal cancer	[47,48,49,50,51]
CD166	lung cancer and colorectal cancer	[42,52]
EpCAM	lung cancer, breast cancer, gastric cancer, liver cancer, and colorectal cancer	[35,52,53,54,55]
CXCR4	breast cancer and gastric cancer	[56,57]
LGR5	breast cancer and gastric cancer	[58,59]
**Intracellular Markers**		
ALDH	lung cancer, breast cancer, gastric cancer, and colorectal cancer	[60,61,62]
Nanog	lung cancer, breast cancer, gastric cancer, liver cancer, and colorectal cancer	[63,64,65,66,67]
Oct-3/4	lung cancer, breast cancer, gastric cancer, liver cancer, and colorectal cancer	[49,64,68,69,70]
SOX2	breast cancer, gastric cancer, liver cancer, and colorectal cancer	[49,64,67,69]
Notch	breast cancer and liver cancer	[50,71]

**Table 2:** Intracellular and extracellular factors leading to cancer cell dormancy.

Factors	Mechanism	Reference
**Intrinsic Factors**		
interferon regulator factor 7 (IRF7)	IRF7 is the master transcription factor responsible for production of type I interferon and transcription of interferon-related genes, suggesting its crucial role in immunosurveillance mediated dormancy.	[23,72]
Spi-C Transcription Factor (SPIC)	Axl regulated by SPIC mediates prostate cancer DTC dormancy in the bone marrow via GAS6/Axl axis; macrophage-expressed gene 1 (Mpeg1) and signal regulatory protein (Sirp) regulated by SPIC are associated with monocytes and macrophages, and these immune-related genes play important role in dormancy maintenance.	[23,73,74]
**Extrinsic Factors**		
TGFβ2	TGFβ2 as ligand binding with TGF-βRIII receptor, initiating p38 MAPK phosphorylate RB protein, which then upregulates p27 and inhibit cancer-cell-cycle progression; TGFβ2 also correlates with GAS6/Axl axis to induce dormancy.	[75]
Bone morphogenetic protein7 (BMP7)	BMP7 binds with BMP receptor 2 (BMPR2) to activate p38 MAPK phosphorylation of RB protein and upregulates cell cycle inhibitor p21 and metastasis suppressor gene NDRG1.	[76]
Leukemia inhibitory factor (LIF)	LIF belongs to belongs to IL-6 cytokine family, binding of LIF with its receptor LIFR controls tumor dormancy possibly through downstream STAT.	[77]
Thrombospondin 1 (TSP1)	TSP1 is a glycoprotein secreted by vascular endothelial cells with anti-angiogenic effect, which is observed to inhibit breast cancer cells proliferation and lead to cell-cycle arrest at G0/G1 phase.	[78]
Osteopontin (OPN)	OPN expressed in endosteal niche could interact with disseminated leukemia cells to induce them into dormancy	[79]
Annexin A2	Annexin A2 upregulates GAS6 and induces cancer cells into dormancy via Annexin A2-GAS6-TAM family (TYRO3, AXL, and MER).	[80]

## Data Availability

All data are available in the main text.
